# Lack of Association between Interarm Systolic Blood Pressure Difference and Coronary Artery Disease in Patients Undergoing Elective Coronary Angiography

**DOI:** 10.1155/2021/6665039

**Published:** 2021-05-06

**Authors:** Mohammad Hosein Mohamadi, Alireza Rai, Mansour Rezaei, Alireza Khatony

**Affiliations:** ^1^Student Research Committee, Kermanshah University of Medical Sciences, Kermanshah, Iran; ^2^Clinical Research Development Center, Imam Ali Hospital, Kermanshah University of Medical Sciences, Kermanshah, Iran; ^3^Social Development and Health Promotion Research Center, Health Institute, Kermanshah University of Medical Sciences, Kermanshah, Iran; ^4^Infectious Diseases Research Center, Imam Reza Hospital, Kermanshah University of Medical Sciences, Kermanshah, Iran

## Abstract

**Aim:**

Peripheral vascular disease (PVD) and coronary artery disease (CAD) are, in many cases, asymptomatic and not usually diagnosed. The timely diagnosis of peripheral vascular diseases can act as an indicator or practical evidence of CAD. Therefore, this study was conducted to determine the relationship between interarm systolic blood pressure difference (IASBPD) and severity and number of coronary artery stenosis.

**Methods:**

The samples in this cross-sectional study consisted of 578 patients who were candidates for coronary angiography, with an average age of 57.5 ± 10.5 years. Patients were classified according to CAD and number and severity of coronary artery stenosis. The relationship between IASBPD and presence or lack of CAD as well as the number and severity of coronary artery stenosis was studied. The sensitivity, specificity, and positive predictive value of IASBPD index were calculated for the detection of CAD using the Kappa coefficient.

**Results:**

There was no statistically significant relationship between IASBPD, CAD, and severity and number of coronary artery stenosis. This index had low sensitivity and predictive value in the diagnosis of CAD and stenosis in coronary arteries in comparison with angiography.

**Conclusion:**

The results showed that the IASBPD index cannot be a valid criterion for the diagnosis of CAD as well as the number and severity of coronary artery stenosis. More studies with larger sample sizes and different designs are needed in this regard to achieve more conclusive results.

## 1. Introduction

Peripheral vascular disease (PVD) and coronary artery disease (CAD) are two major problems of modern medicine [1–4]. By the year 2030, 32.5% of the total mortality is expected to be caused by CVD [1]. Atherosclerosis is a common disease that can affect the arteries. Therefore, CAD and cerebrovascular diseases are commonly seen with PVD [4]. More than one-third of patients have CAD in addition to PVD, and up to a quarter of them also have carotid artery disease. The most important policies used in the clinic to reduce the incidence of CVD and its implications include preventive measures, classification of risk factors, and rapid treatment [1–5]. In addition to clinical examinations and history-taking, invasive procedures such as angiography and noninvasive tests such as Ankle Brachial Index and interarm systolic blood pressure difference (IASBPD) are used to identify and detect PVD [6, 7].

Considering the high prevalence of CVD, presenting and introducing a criterion is very valuable and effective in the screening and diagnosis of PVD and consequently CAD in asymptomatic patients and those who are at the early stages of the disease [4, 6, 7]. It would also help to solve part of the health problems in the society. In this regard, measuring blood pressure from both arms and determining the difference between them is the easiest, cheapest, most useful, and most commonly used noninvasive method to diagnose stenosis and its severity in the subclavian and brachial arteries, which can be used as a criterion for detecting PVD and predicting the existence of CAD [4, 6–8].

The frequency of IASBPD ≥10 mmHg varies from 1–4.0% up to 38.0% depending on the person's individual characteristics [9–11]. Tokitsu et al. (2015) indicated that IASBPD level was higher in patients with CAD than those without CAD. The severity of coronary artery stenosis was also significantly higher in those with IASBPD of greater than 10 mmHg than those with IASBPD of less than 10 mmHg. On the other hand, the probability of future cardiovascular events was significantly higher in people with IASBPD ≥10 mmHg, and the prediction ability of this index was more independent and stronger than the other common risk factors of CVD [11].

In this regard, Clark et al. (2016) showed that the overall mortality and CVD mortality rate were higher in patients with IASBPD ≥10 mmHg than in patients with IASBPD of ≤10 mmHg [9]. Yamamoto et al. (2015) reported that IASBPD was not significantly different between patients with CAD and those without CAD; therefore, IASBPD could not be a predictive factor for the existence and severity of CAD [12]. Given the limitations and contradictory results of available studies, the current study aimed to determine the relationship between IASBPD and CAD in patients undergoing angiography. This study also attempted to determine the sensitivity and specificity of IASBPD in predicting and diagnosing CAD. In this study, we sought to answer the following questions: (1) What is the level of IASBPD in patients undergoing angiography? (2) What is the frequency of severity and number of coronary artery stenosis in patients undergoing angiography? and (3) What is the relationship between the IASBPD and the severity and number of coronary artery stenosis?

## 2. Methods

### 2.1. Sample and Sampling Method

The study population in this descriptive-analytical and cross-sectional study consisted of all patients attending the catheter section of Imam Ali Hospital. This hospital is a subspecialty cardiology center in western Iran, Kermanshah, where usually 500 angiographies are performed monthly. For sampling, about 30% of patients who underwent angiography were enrolled in the study monthly. Patients who are candidates for angiography are admitted to the CCU ward one day before angiography. Sampling was done by convenience sampling method. For this purpose, the researcher referred to the CCU ward on a daily basis and included samples with inclusion criteria. Patients were included if undergoing elective CAG, if it was possible to measure blood pressure from both arms, having systolic blood pressure of 90–200 mmHg, and absence of a history of coronary artery bypass graft, congestive heart failure (based on ejection fraction of less than 30.0%), severe heart valve disease (based on echocardiography), congenital heart disease (based on echocardiography), atrial fibrillation rhythm (based on electrocardiogram), kidney failure (creatinine of more than 2 or performing hemodialysis), systemic inflammatory diseases (fever or evidence of the diseases), systemic thromboembolism (according to patient's history), and pregnancy in female patients. Cancellation of CAG for any reason led to the withdrawal of the sample from the study and its replacement with another patient.

The sample size was calculated based on the study of Tak et al. (2013) [8]. For this purpose, the R-value (proportion of people with IASBPD ≥10 mmHg) was 0.121. Further, P0 as the outcome rate for subjects with IASBPD <10 mmHg and P1 as the outcome rate for subjects with IASBPD ≥10 mmHg were considered to be 0.1 and 0.2, respectively. The first and second type errors were considered to be 0.05 and 0.2, respectively. Considering the high values, a sample size of 578 patients was estimated using the sample size estimation formula for the logistic regression model by PASS software version 11.

### 2.2. Instruments

Data collection tools included a demographic information questionnaire and a data sheet for recording blood pressure and CAG results. To measure blood pressure, the Microlife blood pressure measuring device (Watch BP AFIB model, Switzerland), which was capable of simultaneously measuring blood pressure from both arms, was used. This device has a precision of about ± 3 mmHg, which requires calibration every two years according to the manufacturer's recommendations. In this study, a brand new device with accurate calibration was used.

### 2.3. Data Gathering Method

For data gathering, the researcher attended the angiography department of the hospital and selected the patients who met the inclusion criteria. These patients had been admitted a day earlier to undergo angiography. First, the personal information form was completed by the researcher, and then the blood pressure of both left and right arms was measured by a digital device.

Based on the guidelines, a standard tourniquet (arm) with a width of 12-13 cm and a length of 35 cm was used. A larger and a smaller size tourniquet were also available if needed. The patient's blood pressure was measured and recorded by the researcher the night before the CAG. Patients were positioned in a comfortable position for at least five minutes before measuring their blood pressure, and their arms were placed at the same level as their heart in the center of a bed with a height-adjustable table. Patients were asked not to take stimulants such as tea or coffee half an hour before the blood pressure measurement. The device automatically measured the blood pressure at intervals of up to three times per minute and calculated the blood pressure and its mean, which was based on the calculation of the interarm difference (IAD) in the current study. According to the method of Tokitsu et al. (2015) and Weinberg et al. (2014), blood pressure was measured from both arms, and IASBPD ≥10 mmHg was considered the cut-off point [11, 13]. Based on the studies of Hitaka et al. (2015) and Clark et al. (2016), samples were divided into two groups of IASBPD <10 mmHg and IASBPD ≥10 mmHg based on their IASBPD [9, 10].

**Figure 1:**
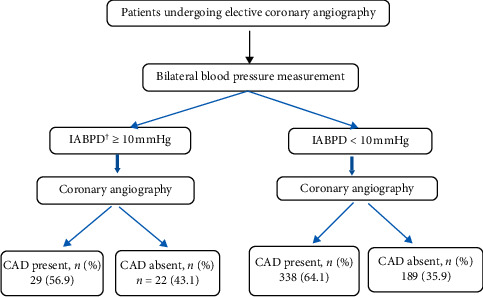
Study flowchart. Note: ^†^interarm systolic blood pressure difference.

CAG was performed by an angiographic specialist using a fluoroscope and X-ray device (Siemens Zee model, manufactured in Germany) with the injection of contrast agent. During the CAG, the number and severity of coronary artery stenosis were determined. After CAG, the severity of the stenosis (in terms of stenosis of the diameter of the vessel) and the number of narrowed vessels (according to the doctor's report) were recorded in the data sheet. Based on the CAG results, the patients were divided into two groups of without CAD (normal coronary artery disease or stenosis of less than 50.0%) and with CAD (with moderate stenosis of 51.0–70.0% and severe stenosis of more than 71.0%). Patients with CAD were divided into three groups according to the number of narrowed vessels, including one, two, and three narrowed vessels. Each of these groups was divided into two subgroups, which included moderate stenosis (stenosis of 51.0–70.0% of the diameter of the vessel) and severe stenosis (stenosis of more than 71.0% of the diameter of the vessel). This process is shown in Figure 1.

### 2.4. Statistical Analysis

Data were analyzed by 16th version of the Statistical Package for Social Sciences (SPSS v.16; SPSS Inc., Chicago, IL, USA) using descriptive statistics (mean and frequency percentage) and inferential statistics (Kolmogorov-Smirnov test, Chi-Square test, kappa coefficient, Spearman, and Pearson correlation coefficients). The Chi-Square test was used to determine the relationship between IASBPD and the number and severity of coronary artery stenosis. To determine the predictive power of IASBPD index in diagnosing CAD (based on stenosis of more than 50.0% in at least one major coronary artery), kappa coefficient was used. *P*-value of less than 0.05 was considered significant.

### 2.5. Ethical Considerations

The KUMS' Ethics Committee approved the study. The goals of the study were explained to the participants and written informed consent was obtained from all of them. The participants were assured about the confidentiality of their personal information and their profile.

## 3. Results

The purpose of this study was to determine the relationship between IASBPD and the severity and number of coronary artery stenosis in patients undergoing CAG. For this purpose, IASBPD was measured, and its predictive power was assessed to detect the presence of CAD. Of the 578 patients who participated in this study, 333 (57.6%) were male. The mean age of the subjects was 57.5 ± 10.5 years, and they had an average body mass index of 27.55. About half of the samples (47.9%) had a history of HTN and 135 patients (23.4%) had diabetes. Moreover, 186 of them (32.0%) had HLP and 144 (25.0%) were smokers. The mean left ventricular ejection fraction was 49.0 ± 8.0%. The results showed that 11.0% (*n* = 27) of women, 9.6% (*n* = 43) of patients aged >50 years, and 6.3% (*n* = 9) of smokers had IASBPD ≥10 mmHg. Finally, 14.1% (*n* = 28) of subjects with LASBP ≥140 mmHg (left arm systolic blood pressure) and 10.8% (*n* = 20) of them with RASBP ≥10 mmHg (right arm systolic blood pressure) had IASBPD ≥10 mmHg (Table 1).

The mean systolic blood pressure (SBP) levels of the right and left arms were 133.3 ± 20.4 and 131.8 ± 20.0 mmHg, respectively. The lowest and highest SBP levels were 90 and 199 mmHg, respectively. The SBP level in the left arm in 198 (34.0%) of the samples was equal to or greater than 140 mmHg, and SBP level in the right arm in 185 (32.0%) of them was equal to or greater than 140 mmHg. A total of 367 patients (63.5%) had at least one narrowed vessel with the stenosis level of ≥50.0%, who were considered to have CAD. In addition, 211 (36.5%) of the samples had no significant stenosis in their coronary arteries. The frequency of IASBPD ≥10 mmHg in patients with three narrowed vessels (with any level of stenosis) was 10.1% (*n* = 14). The Chi-Square test did not show a statistically significant relationship of IASBPD in patients with and without CAD (Table 2).

About 57.0% (*n* = 29) of the samples with IASBPD ≥10 mmHg had CAD and 43.0% (*n* = 22) of them did not have CAD. However, in samples with IASBPD ≤10 mmHg, about 64.0% (*n* = 338) had CAD and 36.0% (*n* = 189) did not have CAD. The Kappa coefficient did not show any correlation between the IASBPD index and the existence of CAD (Table 3).

**Figure 2:**
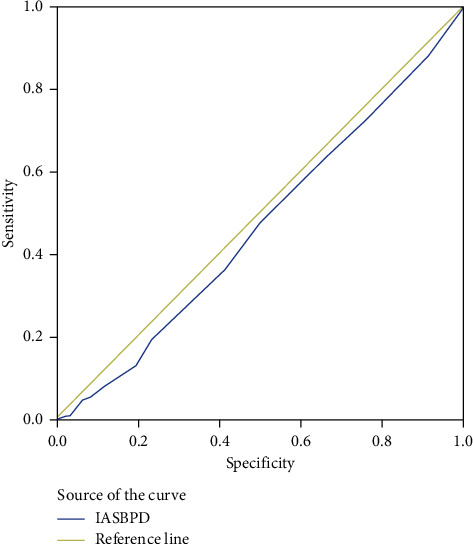
Evaluation of sensitivity and specificity of interarm systolic blood pressure difference (IASBPD) in the diagnosis of coronary artery disease based on ROC curve.

The sensitivity and specificity of the IASBPD ≥10 mmHg index to detect the presence of CAD were 8.0% and 90.0%, respectively. The positive and negative predictive values of the IASBPD ≥10 mmHg were calculated to be 57.0% and 36.0%, respectively, indicating a very low sensitivity and a high specificity. The accuracy of this index, as a test for the detection of CAD, was 38.0% (Table 4) (Figure 2).

The area below the ROC curve represents the total sensitivity and specificity of the IASBPD index in the diagnosis of coronary artery disease compared to angiography. In this figure, the area under the curve is the lowest and indicates the very low predictive value of the IASBPD index compared to angiography, for the diagnosis of coronary artery disease.

Note: IASBPD: interarm systolic blood pressure difference.

## 4. Discussion

In our study, the frequency and mean of IASBPD ≥10 mmHg were 8.8% and 4.2 ± 4.0 mmHg, respectively. In various studies, the frequency of IASBPD ≥10 mmHg has been reported to vary from 1.4% to 38.0% [8, 11]. Aboyans et al. (2007) reported the frequency of 8.8% for IASBPD≥10 mmHg [14], but Clark et al. (2016) reported the frequency of 38.0% for this index [9]. In the above study, blood pressure was measured consecutively using gauged devices. Due to the white coat effect, the amount of blood pressure was higher in the first arm measurement than the second one and might show a higher IASBPD in the consecutive measurement [4, 9]. In our study, we used a device that automatically and digitally measured the blood pressure of both arms simultaneously. In studies that measured blood pressure simultaneously using automated devices, the frequency of IASBPD ≥10 mmHg was less than usual [15].

Our results showed that, among those with IASBPD ≥10 mmHg, approximately 57.0% had CAD and 43.0% did not have CAD, but among those with IASBPD ≤10 mmHg, 64.0% had CAD and 36.0% did not have CAD. Chi-Square test did not show a statistically significant relationship between CAD and IASBPD of less than, equal to, or greater than 10 mmHg. Further, the Kappa coefficient did not show any correlation between the two variables. The frequencies of IASBPD >10 mmHg in patients with and without CAD were 7.9% and 10.1%, respectively.

In the study of Tokitsu et al. (2015), the samples were divided into two groups of patients with CAD and without CAD based on the severity of coronary artery stenosis of at least 75.0% of the vein diameter. The results showed that the frequencies of onset in people with and without CAD were 62.0% and 38.0%, respectively [11]. However, Tokitsu et al. (2015) did not review the number of narrowed coronary arteries. In terms of the frequency of patients with and without CAD, our results are in line with those of Tokitsu et al. (2015). But it should be noted that, in our study, the cut-off point for CAD diagnosis was the presence of at least 50.0% stenosis in one or more major coronary arteries (at least 1.5 mm in diameter), which was different than the cut-off point in the study of Tokitsu et al. (2015). In the study of Yamamoto et al. (2015), the basis for the classification of CAD patients was similar to ours, and the frequencies of patients with and without CAD were 47.0% and 53.0%, respectively. Yamamoto et al. showed that IASBPD had no predictive value for determining CAD [12], which is in line with the results of our study. Moreover, Yamamoto et al. showed the severity of coronary artery stenosis was not related to the absolute level of IASBPD [12].

We did not find a statistically significant relationship between IASBPD ≥10 mmHg and the number and severity of coronary artery stenosis. In fact, IASBPD ≥10 mmHg as an indicator does not have a diagnostic value in predicting CAD. This result can be due to the different effects of atherosclerosis on coronary arteries compared to subclavian arteries or could be due to the weak ability of IASBPD index in identifying stenosis in the subclavian artery. The types of stenosis cause a difference in blood pressure between the two arms that have one-way flow and involve more than 60.0% of the diameter of the artery [4]. In this situation, the blood flow in the affected limb decreases significantly, which causes the loss of blood pressure in the limb, and this decrease can be measured by conventional devices. In the study of English (2001), the frequency of IASBPD was higher in patients with severe stenosis of three vessels (5.3%) than the other groups. The basis for the severity of coronary artery stenosis in the study of English (2001) was the stenosis of more than 50.0% [16]. The reason for the difference in our results and the findings of English can be due to the measurement method of blood pressure from the arms or the study design. Hitaka et al. (2015) did not find a significant relationship between IASBPD and the number and severity of CAD [10]. The results of our study are in line with this study. The results of Tokitsu et al. (2015) study indicated that IASBPD was significantly higher in patients with CAD than those without CAD. Moreover, the severity of coronary artery stenosis was higher in patients with higher IASBPD [11].

We did not find a statistically significant relationship between the frequencies of IASBPD and CAD and the severity and number of coronary artery stenosis, which are not consistent with the results of Tokitsu et al. (2015) [11]. This difference may be due to differences in the design and execution method of the studies or the number of samples. The blood pressure measurement method (consecutive or simultaneous), the patient's position when measuring blood pressure (sitting or lying down), and the type of pressure meter device can affect the level of IASBPD.

In the present study, IASBPD ≥10 mmHg had very low sensitivity and high specificity for the prediction of CAD, indicating the low predictive value of this index for the diagnosis of CAD. Despite extensive searches on credible databases, we did not find any study to determine the sensitivity, specificity, and positive predictive value of the IASBPD index to predict the existence of CAD. English et al. (2001) also used IASBPD index to predict the presence of subclavian artery stenosis. The results showed that IASBPD ≥10 mmHg had low sensitivity (65.0%) and high specificity (85.0%) in the diagnosis of subclavian artery stenosis, with a positive predictive value of 13.0% and a negative predictive value of 99.0%.

English also indicated that IASBPD index can be considered for the prediction of CAD as the subclavian arterial stenosis is accompanied by CAD [17]. The results of our study are not consistent with the results of this study. In the study of Weinberg et al. (2014), the sensitivity and specificity of the IASBPD index in detecting the possible onset of CVD, as a prospective cohort, were investigated among a healthy society, and the results showed that this index had high sensitivity and low specificity in the diagnosis of stenosis in the subclavian artery. Its predictive power had also a high potential for predicting the current and future cardiovascular events [13], confirming the results of the present study. Quiroga et al. (2015) assessed the predictive value of IASBPD ≥10 mmHg in CVA occurrence in renal failure patients. They found a significant correlation between the frequency of IASBPD and cardiovascular morbidity in the future, and IASBPD could be considered an independent indicator for predicting cardiovascular events [16]. Our results are not in line with this study, which could be due to the blood pressure measurement method and the study design.

Our study had some limitations. Several factors affect the accuracy of blood pressure measurements by digital devices, including the low accuracy of device sensors in high or low blood pressures. In our study, people with high blood pressure (greater than 200 mmHg) and very low blood pressure (less than 80 mmHg) were excluded from the study, which could have affected the results. Another limitation was the fact that we could not measure blood pressure from the arms during angiography. We measured the blood pressure of both arms the night before angiography. The last limitation is related to the nature of the convenience sampling method. In this method, because the samples are underrepresentation of subgroups, the probability of selection bias is high, which makes the generalizability of the results limited.

## 5. Conclusion

In our study, there was no statistically significant relationship between IASBPD and the severity and number of coronary artery stenosis. The IASBPD index had lower sensitivity and negative predictive value in detecting the severity and number of coronary artery stenosis than the gold standard of angiography index. Therefore, based on our results, the IASBPD index cannot be used to predict the presence or absence of the coronary artery disease. Given the contradiction between our findings and the results of other existing studies, further studies with larger sample sizes and different designs are necessary.

## Figures and Tables

**Table 1:** Frequency distribution of the demographic variables in terms of IASBPD of less and more than 10 mmHg.

Demographic variables		All (*n* = 578)	IASBPD^†^ <10 mmHg (*n* = 527)	IASBPD ≥10 mmHg (*n* = 51)	*P*-value

Sex, *n* (%)	Male	333 (57.6)	309 (92.8)	24 (7.2)	0.110
	Female	245 (42.4)	218 (89.0)	27 (11.0)	

Smoking, *n* (%)	Yes	144 (24.9)	135 (93.8)	9 (6.3)	0.209
	No	434 (75.1)	392 (90.3)	42 (9.7)	

Hypertension, *n* (%)	Yes	277 (47.9)	247 (89.2)	30 (10.8)	0.103
	No	301 (52.1)	280 (93.0)	21 (7.0)	

Hyperlipidemia, *n* (%)	Yes	186 (32.2)	169 (90.9)	17 (9.1)	0.854
	No	392 (67.8)	358 (91.3)	34 (8.7)	

Diabetes mellitus, *n* (%)	Yes	135 (23.4)	121 (89.6)	14 (10.4)	0.469
	No	443 (76.6)	406 (91.6)	37 (8.4)	

Myocardial infarction, *n* (%)	Yes	141 (24.4)	132 (93.6)	9 (6.4)	0.240
	No	437 (75.6)	395 (90.4)	42 (9.6)	

Cerebrovascular accident, *n* (%)	Yes	27 (4.7)	26 (96.3)	1 (3.7)	0.337
	No	551 (95.3)	501 (90.9)	50 (9.1)	

Peripheral vein diseases, *n* (%)	Yes	24 (4.2)	21 (87.5)	3 (12.5)	0.517
	No	554 (95.8)	506 (91.3)	48 (8.7)	

Age, (yrs)	<50	128 (22.1)	120 (93.8)	8 (6.3)	0.245
	≥50	450 (77.9)	407 (90.5)	43 (9.6)	

Body mass index (kg/m^2^)	<30	422 (73.0)	393 (93.1)	29 (6.9)	0.007
	≥30	156 (27.0)	134 (85.9)	22 (14.1)	

LASBP^‡^ (mmHg)	<140	380 (65.7)	357 (93.9)	23 (6.1)	0.001
	≥140	198 (34.3)	170 (85.9)	28 (14.1)	

RASBP^‡‡^ (mmHg)	<140	393 (68.0)	362 (92.1)	31 (7.9)	0.248
	≥140	185 (32.0)	165 (89.2)	20 (10.8)	

^†^interarm systolic blood pressure difference, ^‡^ left arms systolic blood pressure, ^‡‡^ right arms systolic blood pressure.

**Table 2:** Frequency distribution of the severity and number of coronary artery stenosis in terms of IASBPD of less and more than 10 mmHg.

Number of vessel stenosis cases	Severity of vessel stenosis	Total (*n* = 578)	IASBPD^‡^ <10 mmHg (*n* = 527)	IASBPD≥10 mmHg (*n* = 51)	*P*-value

Single vessel disease	Moderate^†^, *n* (%)	44 (7.6)	42 (95.5)	2 (4.5)	0.834
	Sever^††^, *n* (%)	54 (9.3)	52 (96.3)	2 (3.7)	
	Sum^*θ*^, *n* (%)	98 (17.0)	94 (95.9)	4 (4.1)	

Two-vessel disease	Moderate, *n* (%)	54 (9.3)	52 (96.3)	2 (3.7)	0.100
	Sever, *n* (%)	76 (13.1)	67 (88.2)	9 (11.5)	
	Sum, *n* (%)	130 (22.5)	119 (91.5)	11 (8.5)	

Three-vessel disease	Moderate, *n* (%)	18 (3.1)	15 (83.3)	3 (16.7)	0.319
	Sever, *n* (%)	121 (20.9)	110 (90.9)	11 (9.1)	
	Sum, *n* (%)	139 (24.0)	125 (89.9)	14 (10.1)	

^†^moderate stenosis = 50.0% up 70.0%, ^††^ severe stenosis = >70%, *θ* sum : moderate stenosis + severe stenosis.

**Table 3:** Correlation between the existence of coronary artery diseases and IASBPD^†^ of less and more than 10 mmHg.

		All (*n* = 578)	IASBPD <10 mmHg (*n* = 527)	IASBPD ≥10 mmHg (*n* = 51)	Kappa coefficient

Coronary artery diseases, *n* (%)	Yes	367 (63.5)	338 (64)	29 (57)	−0.019
	No	211 (36.5)	189 (36)	22 (43)	

^†^interarm systolic blood pressure difference.

**Table 4:** The sensitivity and specificity of the IASBPD ≥10 mmHg index to detect the presence of coronary artery diseases.

	Sensitivity (%)	Specificity (%)	Positive predictive value (%)	Negative predictive value (%)	Accuracy (%)	Test result	Kappa coefficient

IASBPD^†^ ≥10 mmHg	8.0	90.0	57.0	36.0	38.0	Χ^2^ *=* 1.061 *P*=0.303	−0.019

^†^interarm systolic blood pressure difference.

## Data Availability

The data are available by contacting the corresponding author.
